# Health‐related quality of life among Ethiopian pregnant women during COVID‐19 pandemic

**DOI:** 10.1002/brb3.2045

**Published:** 2021-01-27

**Authors:** Aman Dule, Mohammedamin Hajure, Mustefa Mohammedhussein, Zakir Abdu

**Affiliations:** ^1^ Department of Psychiatry College of Health Science Mettu University Mettu Ethiopia

**Keywords:** COVID‐19 fear, Ethiopia, pregnancy, quality of life

## Abstract

**Objective:**

To explore the quality of life and its association with perceived social support and pandemic fear among pregnant women.

**Methods:**

Cross‐sectional study was conducted among 384 pregnant mothers at southwest Ethiopia from August 1, 2020, to August 15, 2020. Participants were included by consecutive sampling. Quality of life was assessed by short version of WHO quality of life. Social support and fear of COVID‐19 were evaluated by Multidimensional Scale of Perceived Social Support (MSPSS) and fear of COVID‐19 scale (FCoV‐19S), respectively. Data were analyzed by SPSS version 23.0. Frequency and percentage for categorical variables, and mean ± *SD* for continuous variables were calculated. Independent sample *t* test and ANOVA were employed to compare the groups of normally distributed variables. Multiple regressions were performed, and Pearson correlation (*r*) was used to explore the relationships. Statistical significance was declared at *p* < .05, and 95% CI was calculated.

**Results:**

The mean age of the participants was 31.3 ± 7.7, and 40.4% of them were between the ages of 25–34. The mean scores of participants those living with <5 family members and those living with ≥5 family members were significantly different [*t*(382) = 3.09, *p* = .002]. Participants with primary education have significantly high mean score of WHOQOL‐BREF from those participants with no formal education (*p* = .028, MD = −1.9). Moderate negative correlation was observed between the scores of WHOQOL‐BREF and FCoV‐19S. All the components of MSPSS had positively associated with WHOQOL‐BREF score at significant level. On final model, FCoV‐19S score has uniquely accounted for 19.4% of variance in WHOQOL‐BREF.

**Conclusions:**

Perceived social support has positively linked to QOL among pregnant women during COVID‐19 pandemic. Pandemic‐related fear has negative association with QOL and may be considered independent contributor of decreased quality of life in this population.

## Graphical abstract

This study was aimed to explore the quality of life among pregnant women during corona virus disease 2019 (COVID‐19). Statistics such as Independent samples *t*‐test, One‐way analysis of variance (ANOVA), Pearson's correlation and standard multiple regression were employed. The mean age of the participants was 31.3 ±

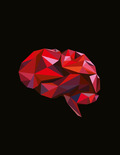

7.7 and perceived social support has positive effect on quality of life among pregnant women.

## 1 INTRODUCTION

Since its onset, coronavirus disease 2019 (COVID‐19) pandemic has posed unpredicted psychosocial burdens which had affected all individuals globally. After its outbreak, inappropriately exaggerated coverage of traditional media concerning the pandemic had elevated psychological disturbances of the people (Olatunji et al., 2020). Specifically, in those communities with weak health‐care system such as Sub‐Saharan countries, the pandemic has fueled high dread (Mohammed et al., 2020).

Being one of these countries, Ethiopia is also at the edge of losing its progression of health‐care system which made over the last decades unless the control of this pandemic has ensured (Biadgilign & Yigzaw, 2020). Immediately after the report of first case on March 13, 2020, Ethiopia has adopted protective measures on March 16th energized by the stress of dealing with the outbreak in fragile health‐care system of the country (Shigute et al., 2020). In strengthening the measures, the country has declared the state of emergency and prepared national guideline to implement a uniform preventive protocol in the month of April (FMOH, 2020b). Despite these, the case is alarmingly increasing in the country. As of December 20, Ethiopia has reported 119,951 confirmed cases (which put the country at the top in East Africa) and a total death of 1,853 with case fatality rate of 1.5 (FMOH, 2020a).

Pandemic outbreak has known to causes collective experience of psychopathological outcomes (Pariente et al., 2020). Particularly, women were subjected to mental health disturbances and lowered global quality of life during the current pandemic (Pulvirenti et al., 2020). Specifically, emotional and physical changes during the transition state of pregnancy could affect the quality of life in pregnant mothers (Lagadec et al., 2018). They are at high risk for mental well‐being instability, and the proportion is high in developing countries (Couto et al., 2009). On the other hands, radically modified habitual routines of the people as a result of protective measures have substantially affected health‐related quality of life (HRQoL) among pregnant women (Bivi et al., 2020). For instance, previous study reported that the interrupted regular follow‐ups among pregnant women during COVID‐19 pandemic have affected their mental well‐beings which probably play a crucial role in disturbed quality of life (Zeng et al., 2020). Supportive finding revealed that physical component of QOL was decreased throughout pregnancy and has associated with primiparity and pregnancy‐related complications (Couto et al., 2009). During COVID‐19 pandemic, QOL has moderately disturbed in which unemployed and older participants were highly affected (Ping et al., 2020; Samlani et al., 2020).

In promoting the well‐beings and decreasing the adverse outcomes in pregnant mothers, the importance of social support is increasing (Abdollahpour et al., 2015) and it has paramount effects in reducing psychological distress during pregnancy (Shishehgar et al., 2015). Studies had conveyed that strong social support during pregnancy has positive effects in combating maternal depression and linked to improved quality of life (Elsenbruch et al., 2007; Lau et al., 2014). During COVID‐19 pandemic, social support has identified as having negative effect against maternal anxiety (Yue et al., 2020) and perceived stress (Alan et al., 2020) in pregnant mothers. Supportively, during current pandemic, the likelihood of anxiety and depressive symptoms was potentially increased in pregnant women with poor support from family members (Molgora & Accordini, 2020) and greater life satisfaction was associated with better perceived social support (Yu et al., 2020).

Although the optimal anxiety during stressful event is a common reaction, unpredicted onset of COVID‐19 pandemic has brought uncertainty and fear among people. Especially, life‐changing circumstance like pregnancy gives a way to anticipated fear and experience of negative emotions during current pandemic (Souto et al., 2020). In addition to worrying about own health, the expectation that COVID‐19 could cause structural abnormalities to the fetus and induces preterm birth were intensified the fear of COVID‐19 among pregnant mothers (Mappa et al., 2020). Apart from health concerns, disruptions in sources of income as a result of lockdown and economic instability were another sources of fear for pregnant women which could disturb their QOL (Kajdy et al., 2020). Study revealed that the effects of COVID‐19 fear among pregnant women had associated with stress, depression, disturbed QOL and extends to suicidal ideation (Ahorsu, Imani, et al., 2020).

The far‐reaching routine changes during the pandemic along with elevated fear could cause substantial decrease in QOL among pregnant women. Thus, knowing the extents of pandemic‐related fear and highlighting the importance of social support are so vital to maintain well‐beings and QOL. In spite of this, studies were lacking so far in Ethiopia to our knowledge to evaluate the quality of life during pregnancy amid current pandemic. Considering this, the main purpose of current study was to assess the QOL among pregnant mothers during COVID‐19 and its association with social support and fear of the pandemic. In light of this objective, the findings of this study will help as baseline for future studies of similar topics. Additionally, it will contribute an input for clinical practitioners those working with this population to provide evidence‐based services. Furthermore, the result of the current study will assist health‐care planners and policy makers in the context of this pandemic.

### 1.1 Hypotheses of the study

H_0_: All respondents with different characteristics equally experienced quality of life in the era of COVID‐19 pandemic.

H_1_: All respondents with different characteristics not equally experienced quality of life in the era of COVID‐19 pandemic.

## 2 MATERIALS AND METHODS

### 2.1 Study setting and participants

This study was conducted among 384 pregnant mothers who were on antenatal care (ANC) follow‐up at health institutions of Mettu town. Mettu is a zonal town which located 600 kilometers away from Addis Ababa, the capital city of Ethiopia to southwest. The town has one referral hospital and two medium clinics which giving ANC services from which the study participants were recruited.

### 2.2 Study design and period

Facility‐based cross‐sectional study was carried out from August 1, 2020, to August 15, 2020.

### 2.3 Inclusion and exclusion criteria

Pregnant mothers who were on regular follow‐up and those who had achieved basic ANC services were included. Those women with any limitation that might hinder them from replying to the interview and identified with high‐risk pregnancy were excluded from the study.

### 2.4 Sample size and sampling procedures

Sample size was calculated using single population proportion formula by considering 95% confidence interval, 5% margin of error, and estimated proportion of 50%. Accordingly, calculated sample (384) was proportionally allocated to three health institutions mentioned above in accordance with the flow of their pregnant mothers on ANC follow‐ups. Finally, those participants who fulfilled the inclusion criteria were included by consecutive sampling technique until the intended number was achieved.

### 2.5 Data collection procedures and instruments

Face‐to‐face interview method was used to collect data by keeping the minimum distance of one meter (1 m) and using necessary protective materials like face mask. Original English versions of questionnaires were initially translated into local languages (Afan Oromo and Amharic). Then, it was converted back to English by linguistic professional to ensure consistency. The questionnaires had contained socio‐demographic characteristics, clinical factors, and questions to assess quality of life, social support and fear of COVID‐19 among participants. Socio‐demographic and clinical characteristics were obtained from participants and from their medical record during ANC follow‐ups.

Quality of life was considered as dependent variable and assessed by short version of WHO quality of life (WHOQOL‐BREF). It contains a total of 26 items from which 24 items categorized into four domains (physical, psychological, social relationships, and environmental). The remaining two questions were scored individually to assess perception of person about their quality of life and overall health (WHO, 1998). The raw score of the individual items should be transformed to the range of 4 to 20 and then to comparable ranges from 0 to 100 (WHO, 1996). In the current study, we had used the score from 4 to 20 for simplicity. Therefore, four domains were scored from 4 to 20 to give a total of 16 to 80 points. The remaining two individual items were scored on Likert scale from 1 to 5 and then added to domain score to yield overall of 18 to 90 points and the higher score indicates better quality of life. The tool was used in pregnant women in different settings (Vachkova et al., 2013; Webster et al., 2010). It has validated in Ethiopian context with excellent internal consistency (Cronbach's alpha = 0.93) (Tesfaye et al., 2016). In current study, the Cronbach's alpha of this tool was 0.90.

The revised Multidimensional Scale of Perceived Social Support (MSPSS) was employed to evaluate social support. It was developed by Zimet et al. (1988) and identifies three sources of support (friends, family, and significant others). Each domain has four items which scored on 7–point Likert scale from 1 (very strongly disagree) to 7 (very strongly agree). Overall, the higher the score, the better the perceived social support is (Başol, 2017). The tool was widely used among pregnant mothers (Aşçi and Gökdemir, 2019; Saeieh et al., 2017; Stewart et al., 2014) and has validated in one Africa country (Cronbach's alpha = 0.916) (Stewart et al., 2014). In Ethiopian context, the tool has not validated yet and the Cronbach's alpha was 0.87 in current study.

Coronavirus disease (COVID‐19) associated fear was assessed by fear of COVID‐19 scale (FCoV‐19S). It was developed and validated among general population, and it has seven items which scored on 5‐point Likert scale from 1(strongly disagree) to 5 (strongly agree). It gives the total score from 7 to 35 in which the higher score indicates greater fear of COVID‐19 (Ahorsu, Lin, et al., 2020). Although it was after our study period, currently this tool has validated in Ethiopia and has good internal consistency (Cronbach's alpha = 0.873) (Aman et al., 2020), and the Cronbach's alpha was 0.907 in our study.

### 2.6 Statistical analyses

SPSS version 23.0 (IBM, Armonk) was employed for data analysis. Frequencies and related percentages were used to present categorical variables, and mean ± *SD* was expressed for continuous variables. To compare the groups of normally distributed variables, independent sample *t* test and one‐way ANOVA were employed and post hoc analysis was performed using Tukey HSD test. Multiple regressions analysis was performed and Pearson correlation (*r*) was used to define direction and strength of relationship among variables. Coefficient of determination (*R* ^2^) was calculated to express model. All multiple regression assumptions were checked and no violation detected. Linearity and multicollinearity were checked by scatter plot and variance inflation factor (VIF), respectively. Independency of residuals was tested by Durbin–Watson, and p‐p plot was employed to check normality. Statistical significant was declared at *p* < .05, and 95% CI was calculated.

### 2.7 Ethical consideration

Written and informed consent was signed by all participants of the study and ethical clearance was gained from the ethical review committee of Mettu University and all research protocol and regulations were followed as per states of ethical committee of the university.

## 3 RESULTS

### 3.1 Characteristics of study participants

The intended number of participants was achieved throughout the study period without attrition rate, and all collected data were fully analyzed without any missing values. The mean age of the participants was 31.3 ± 7.7, and 40.4% of them were between the ages of 25–34. More than one‐fourth of the women were government employees, majority (59.4%) of them were multiparous and about one‐third of women were at third trimester. More than half (59.1%) of the women were currently living in urban area and of total participants, and only 13% of them had reported any pregnancy‐related complication (Table 1).

**TABLE 1:** Socio‐demographic characteristics of study participants (*n* = 384)

Variables	Category	Frequency (%)	*M* ± *SD*
Age	18–24	88 (22.9)	49.4 ± 5.2
	25–34	155 (40.4)	50.5 ± 4.8
	35–44	141 (36.7)	50.1 ± 4.7
Educational status	No formal education	104 (27.1)	49.3 ± 5.3
	Primary	104 (27.1)	51.2 ± 4.0
	Secondary	113 (29.4)	49.8 ± 5.2
	Above secondary	63 (16.4)	50.2 ± 4.8
Occupation	Government employee	103 (26.8)	49.5 ± 4.7
	Self employed	126 (32.8)	50.3 ± 5.1
	House wife	155 (40.4)	50.3 ± 4.8
Parity	Primiparous	156 (40.6)	50.2 ± 4.4
	Multiparous (2 or more)	228 (59.4)	50.0 ± 5.2
Trimester	First	149 (38.8)	49.3 ± 4.6
	Second	105 (27.3)	51.5 ± 5.2
	Third	130 (33.9)	49.9 ± 4.8
Residency	Urban	227 (59.1)	50.1 ± 4.7
	Rural	157 (40.9)	50.1 ± 5.7
Pregnancy complication	Yes	50 (13.0)	50.9 ± 5.8
	No	334 (87.0)	50.0 ± 4.7
Family size	Less than 5	220 (57.3)	50.8 ± 5.0
	Five and above	164 (42.7)	49.2 ± 4.6

Abbreviations: *M*, mean; *SD*, standard deviation.

### 3.2 Comparisons of sample means

To compare the mean score of quality of life for parity, current residence, pregnancy‐related complications, and family size, an independent samples *t* test was employed. Accordingly, only family size was showed significant mean difference. The mean scores of participants those living with <5 family members [(*M* = 50.8, *SD* = 5.0) and those living with ≥5 family members (*M* = 49.2, *SD* = 4.6) were significantly different *t*(382) = 3.09, *p* = .002] at small effect size (eta squared = 0.02) (Table 2).

**TABLE 2:** Independent samples *t* test of quality of life^a^ score for dichotomized predictors

Predictors	*F*	*t*‐Value	*df*	Sig.	Mean difference	Effect size
Parity						
Primiparous	5.5	0.35	363.2	.73	0.17	<0.001
Multiparous						
Residency						
Urban	0.45	0.13	382	.90	0.07	<0.001
Rural						
Complication						
Yes	4.1	1.28	382	.20	0.95	<0.001
No						
Family size						
<5	0.39	3.09	382	**.002** ^b^	1.54	0.02
≥5						

^a^ Quality of life was measured by short version of WHO quality of life (WHOQOL‐BREF) and higher score indicating higher quality of life.

^b^ Statistical significance was considered at *p* < .05, and significant value was bolded.

One‐way analysis of variance was conducted between groups to explore the effects of age, educational status, occupation, and gestational age on participants’ quality of life. No significant differences were observed among age groups and occupational status. However, statistically significant difference was obtained among the scores of participants’ educational status [*F*(3, 380) = 2.8, *p* = .04, eta squared = 0.02] and trimester [*F*(2, 381) = 7.0, *p* = .001, eta squared = 0.04]. Post hoc analysis of Tukey HSD test indicated that the mean score of participants with no formal education (*M* = 49.3, *SD* = 5.3) and those with primary education (*M* = 51.2, *SD* = 4.0) was different at significant level (*p* = .028, MD = −1.9). On the other hands, mothers at first trimester (*M* = 49.3, *SD* = 4.6) had different mean score from those mothers who were at second trimester (*M* = 51.5, *SD* = 5.2). In the similar manner, statistically significant difference of mean score was existed between those women at second trimester and third trimester (*p* = .022 and MD = 1.7) (Table 3).

**TABLE 3:** One‐way analysis of variance for the score of quality of life^a^ for participants’ age, educational status, occupation, and trimester stage

Predictors	Sum of squares	*df*	Mean square	*F*	*p*‐Value	Effect size
Age						
Between groups	60.9	2	30.4	1.3	.28	0.007
Within groups	9,074.6	381	23.8			
Total	9,135.4	383				
Educational status						
Between groups	199.9	3	66.6	2.8	.04^*^	0.02
Within groups	8,935.6	380	23.5			
Total	9,135.4	383				
Occupation						
Between groups	52.8	2	26.4	1.1	.33	0.006
Within groups	9,082.6	381	23.8			
Total	9,135.4	383				
Trimester						
Between groups	325.7	2	162.9	7.0	**.001** ^b^	0.04
Within groups	8,809.7	381	23.1			
Total	9,135.4	383				

Abbreviation: *df*, degree of freedom.

^a^ Quality of life was measured by short version of WHO quality of life (WHOQOL‐BREF) and higher score indicating higher quality of life.

^b^ Statistical significance was considered at *p* < .05, and significant value was bolded.

### 3.3 Correlation analysis

To explore the relationships between outcome variable (QOL) and independent variables, Pearson correlation coefficient (*r*) was employed. Accordingly, moderate negative correlation was observed between the scores of WHOQOL‐BREF and FCoV‐19S (*r* = −.45, *p* < .001). The scores for all components of Multidimensional Scale of Perceived Social Support (MSPSS) had shown positive relationships with WHOQOL‐BREF score at significant level (*r* ranged from .11 to .23) (Table 4).

**TABLE 4:** Pearson's correlations analysis for the scores of WHOQOL‐BREF, FCoV‐19S, and MSPSS components

Predictors	1	2	3	4	5	Sign.
1. Global WHOQOL‐BREF score	–					
2. Support from family	.11	–				0.029
3. Support from friend	.23	.74	–			<0.001
4. Support from significant others	.18	.59	.66	–		<0.001
5. FCoV–19S score	−.45	−.02	−.08	−.14	–	<0.001

Abbreviations: FCoV‐19S, fear of COVID‐19 scale; MSPSS, Multidimensional Scale of Perceived Social Support; WHOQOL‐BREF, WHO quality of life short version.

### 3.4 Multiple regression analysis

Multiple regression analysis was carried out for each component of MSPSS, overall score of MSPSS and FCoV‐19S to decide which variable could predict best the QOL from WHOQOL‐BREF. Consequently, supports from family and from friends were significant predictors of QOL among MSPSS components and accounted for 1.0% and 3.2% total variance of WHOQOL‐BREF, respectively. The overall scores of MSPSS and FCoV‐19S had predicted a total of 23.3% variance in WHOQOL‐BREF, and 19.4% of the variance was uniquely accounted by FCoV‐19S score. (Table 5).

**TABLE 5:** Multiple regression analysis to predict quality of life^a^ from the components of MSPSS, overall score of MSPSS and FCoV‐19S

Predictors	Standardized estimation (*β*)	*t*	*p‐*Value	Accounted variance (%)	95% CI
MSPSS^b^ components					
Support from family	−0.15	−2.0	**.045**	1.0	−0.53, −0.01
Support from friends	0.30	3.6	**.000**	3.2	0.19, 0.64
Support from significant others	0.08	1.1	.265	–	−0.10, 0.36
Model summary*: R* ^2^ = 6.5%, *F* = 8.9, *df* = 3, *p* < .001					
MSPSS	0.16	3.6	**.000**	2.6	0.04, 0.15
FCoV‐19S^c^	−0.44	−9.8	**.000**	19.4	−0.57, −0.38
Model summary*: R* ^2^ = 23.3%, *F* = 57.8, *df* = 2, *p* < .001					

Note

Statistical significance was considered at *p* < .05, and significant value was bolded.

Abbreviation: CI, Confidence Interval.

^a^ Quality of life was measured by short version of WHO quality of life (WHOQOL‐BREF) and higher score indicating higher quality of life.

^b^ MSPSS, Multidimensional Scale of Perceived Social Support (Higher score indicating high perceived social support from family, friends and significant others).

^c^ Fear of COVID‐19 scale—High score implies greater fear of COVID‐19.

## 4 DISCUSSION

In current cross‐sectional study, we had tried to assess the relation of QOL with socio‐demographic characteristics, perceived social support and fear of COVID‐19 among pregnant women and it was assumed to be the first study nationally. The result revealed that middle‐aged women had scored the best average value (50.5 ± 4.8) on the score of WHOQOL‐BREF scale and followed by older women (50.1 ± 4.7). The score of younger women was the lowest (49.4 ± 5.2) although the mean differences between the groups were not reached the significant level (*p* = .28). This finding was inconsistent with previous study (Mazúchová et al., 2018) in which younger‐aged women had scored highest value. This probably due to the difference in employed tools, standard of living between study areas and study year. On the other hands, most of the younger participants were primiparous and possibly they experienced pregnancy as stressful event especially with synergistic effects of current pandemic crisis.

The mean score of WHOQOL‐BREF for those women on second trimester was highest and significantly different from the score of those women who were on first and third trimester. This finding was in agreement with previous study (Hitimana et al., 2018) and inconsistent with others (Lagadec et al., 2018; Mazúchová et al., 2018) in which women at early pregnancy had scored best on quality of life scale. This may be due to the fact that factors like increased weight gain and concern about labor during third trimester and temporary symptoms like nausea and vomiting at the time of first trimester could affect the quality of life among pregnant mothers (Zarajczyk, 2019).

On correlation analysis, our study explored that the scores of all MSPSS components had significant positive association with WHOQOL‐BREF score and the score of overall MSPSS scale has explained 2.6% of unique variance (*t*‐value = 3.6, *p* < .001) in WHOQOL‐BREF scale. This finding is in line with previous studies (Elsenbruch et al., 2007; Shishehgar et al., 2013; Lau et al., 2014; Yu et al., 2020) which had stated that pregnant mothers with good social support had better quality of life. This can be explained by the fact that support and encouragement during pregnancy could help women to decrease negative emotions which in turn improve quality of life.

In our study, we had also examined the association of current COVID‐19 related fear with QOL among pregnant mothers. As expected, the fear of this pandemic has moderate negative effect (*t*‐value = −9.6, *p* < .001) on QOL and 19.4% variance of WHOQOL‐BREF has explained by FCoV‐19S score. This probably supported by the fact of multidimensional impacts from the current pandemic in general, and specifically, the effect can be worsen in pregnant mothers who had additional burden of physiological changes during pregnancy. Moreover, the pandemic has introduced different psychosocial disturbances including mental health problems such as anxiety, stress, and depression that could disturb QOL in many aspects. There were previously existing supportive evidences to this finding (Li et al., 2020; Micelli et al., 2020; Nguyen et al., 2020; Shacham et al., 2020; Zhang & Ma, 2020) which revealed that pandemic‐related fears had identified to affect the life quality of pregnant women.

While conducting the current study, we had identified some limitations. The first limitation was its cross‐sectional nature which hindered the inference of causality. On the other hand, conducting this study at single setting along with consecutive sampling could limit the generalization of the findings. Although we had assessed for occupational status, the details of specific income level were not collected which can affect the living situation of the participants. Furthermore, relatively low value of *R*‐square (23.3%) indicated that more variables should be included to increase the predictive capacity of this model. To address these limitations, further studies are required in the future. Despite the mentioned limitations, our study was thought to be the first at national level and some impacts of the current pandemic were highlighted. As such, it supposed to raise different hypotheses during pandemics in related topics. Moreover, using the standardized tools to assess outcome variable and major independent variables was the other strength of this study.

## 5 CONCLUSIONS

In agreement with our alternative hypothesis, the life quality of pregnant women with different characteristics has not affected equally during COVID‐19 pandemic. As indicated in the current study, perceived social support has positively associated with QOL among pregnant women which should be encouraged to get better quality of life and satisfaction during this physiological change. During this pandemic crisis, getting psychological and emotional support from family member, friends, and significant others could help pregnant mothers to cope with the effects of pandemic and get better outcome in their quality of life. From the finding, we had also concluded that the current pandemic has imposed great fear among pregnant women which leads to decreased quality of life. Enhancing the awareness and showing the real picture of the illness and educating preventive behaviors along with good social support could help this population in coping with the pandemic associated fears and in turn boost quality of life.

The findings of this study had research and practical implications in showing one or more ways in which COVID‐19 pandemic can affects QOL during pregnancy. In light with this, knowing the effects of pandemic fear and the importance of social support could help service providers and health planners in encouraging support and combating pandemic‐related fears. From the abovementioned points, maintaining social support and improving the understanding of the current pandemic could be helpful to improve QOL for pregnant women.

## CONFLICT OF INTEREST

The authors declare that they have no conflict of interest.

## AUTHOR CONTRIBUTIONS

All authors had contributed substantially in conceptualizing, designing, data collection, analysis, and interpretation of data; drafting, reviewing, and approving the final version to be published; have agreed to submission to this journal and to be accountable for all aspects of this work. AD and ZA highly contributed in conception, designing, and critical revision for important intellectual content. MH and MM greatly contributed to acquisition of data, data analysis and interpretation and manuscript drafting.

### PEER REVIEW

The peer review history for this article is available at https://publons.com/publon/10.1002/brb3.2045.

## Data Availability

The data supporting the findings are available with corresponding author on reasonable request.
